# Social Networking Services as a Tool for Support of Mothers: A Literature Review

**DOI:** 10.1089/whr.2022.0026

**Published:** 2022-11-14

**Authors:** Tomoko Oto, Jun Watanabe, Yasunobu Ito, Kazuhiko Kotani

**Affiliations:** ^1^ Hosei University Center for University-Community Collaboration, Hosei University, Machida, Japan.; ^2^ Division of Community and Family Medicine, Jichi Medical University, Shimotsuke, Japan.; ^3^ School of Knowledge Science, Japan Advanced Institute of Science and Technology (JAIST), Nomi, Japan.

**Keywords:** information technology, internet, mothers, social media, social support

## Abstract

**Background:**

Difficulties experienced by mothers in raising their children can be resolved using social networking services (SNSs). Being aware of issues associated with SNSs in such situations may be useful for supporting mothers. We herein review the issues associated with using SNSs to support mothers.

**Methods:**

This review was conducted using an electronic search engine to search for articles that described issues associated with using SNSs to support mothers, and which were published up to August 2022.

**Results:**

After screening, a total of 19 articles were included. We thematically categorized the contents into three major issues associated with using SNSs for support of mothers: (1) issues on the management side, (2) issues on the user side, and (3) social and environmental issues. The mainly discussed issues were the safety of using SNSs and/or securing and training human resources on the management side, busyness of mothers on the user side, as well as sociocultural and communication environment-related limitations as social and environmental issues.

**Conclusions:**

The issues we detected would aid in developing the use of SNSs as a tool to support mothers. Further research on these issues is needed.

## Introduction

Life events, such as pregnancy, childbirth, and childcare, occasionally place negative stress on mothers' mental health; for instance, mothers tend to become depressed^1^ and to be isolated from diverse social networks.^2^ Social isolation lowers a mother's self-esteem, which in turn worsens her own health and thereby the health of her children.^3^ Postpartum depression (the incidence of which is estimated to be 12%–14%^4,5^) is a mental disorder that can lead to maternal suicide. Support for the health and well-being of all mothers and children is thus recognized to be a matter of global importance.^6^ However, with the exception of Northern Europe, the rate of men taking parental leave remains low worldwide; thus, the physical and mental burden of childcare on mothers remains high.^7^

For this reason, medical institutions offer some support services (*e.g.,* childcare and postpartum breastfeeding guide) for mothers. This helps mothers gain knowledge and skill in childcare. When considering support for mothers during child-rearing, they may also need comprehensive nonmedical support from their families and other mothers. In addition, due to COVID-19 pandemic, mothers may be encouraged to avoid face-to-face connections and support. As a result, mothers are not able to help each other or receive appropriate information. Thus, online remote support has garnered attention over face-to-face support.

Currently, various social networking services (SNSs) have been developed, and research on supporting mothers with SNSs and the communities has been conducted in many countries. In general, such social networking and online networks have a positive impact on mothers.^3,8–15^ In particular, for socially vulnerable adolescent mothers, social networking sites are proper tools for receiving social support, including information on child-rearing.^9,10,13^ In relation to mental health, communication among mothers on social networking sites can reduce maternal stress^9,12^ and increase maternal self-esteem.^9^ Social networking sites are also reported to reduce mothers' sense of loneliness during child-rearing.^3^

Knowing the general issues related to SNSs may be useful for supporting mothers. However, such issues have not been summarized. This study therefore examined the issues associated with using SNSs as tools to support mothers in the medical field.

## Methods

This review was conducted using an electronic search engine to identify published articles based on the following search keywords: mother, online social network, social networking service, SNS, social media. A search was then conducted using PubMed and CENTRAL for articles published through to August 3, 2022. Boolean operators were included in search terms, using truncation to further broaden results and include plurals or similar terminology: “Mothers”[Mesh] OR “Mother”[tiab] OR “mothers”[tiab]) AND (“Online Social Networking”[Mesh] OR “Online Social Network”[tiab] OR “Online Social Networking”[Mesh] OR “Online Social Networking”[tiab] OR “Social Networking Ser-vices “[tiab] OR “Social Networking Sites”[tiab] OR “SNS”[tiab] OR “ Social Media”[tiab].

The inclusion criteria were articles that evaluated the effects of SNS on maternal health in mothers. Articles that reported on the term “SNS” (the abbreviation) with a different meaning from social networking services (*e.g.,* sympathetic nervous system^16^), articles in which the study subjects were not mothers (*e.g.,* midwives^17,18^), which focused on the development of a system (*e.g.,* the process of creation of messages^19–21^), and which provided an overview of SNSs (*e.g.,* the actual states of use of SNSs in pregnancy^22^) were excluded.

**FIG. 1.**
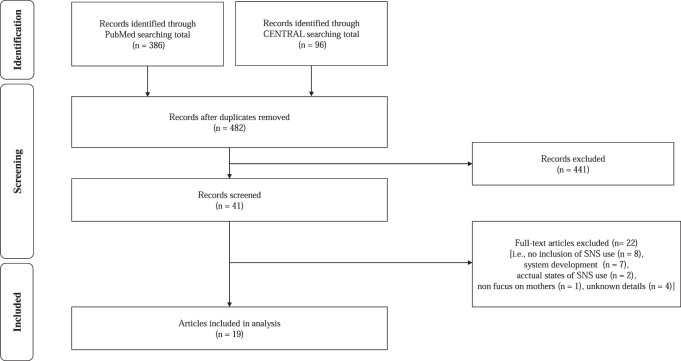
Flowchart of the study selection process. SNS, social networking service.

Figure 1 shows the flowchart of the process. First, titles and abstracts were screened according to the inclusion/exclusion criteria. Second, full-text screening was conducted. Finally, selected articles were carefully read to identify factors that were issues associated with using SNSs in support. Quantitative studies using all methodologies were included in this review.

Issues associated with using SNSs to support mothers were identified and then categorized with reference to the methods using Nvivo^23,24^: First, selected articles were imported into Nvivo (ver. 20.5.0). Next, sentences from the text of the selected articles that were determined to be issues associated with using SNSs to support mothers were extracted and given appropriate node names to indicate their content. This process was repeated, and multiple nodes were created. After node extraction was completed, nodes with similar contents were grouped together. In this process, another author cross-checked for consistency in the coding approach and interpretation. Finally, the issues were summarized into the major categories of issues. This review method with its thematic categorization was similarly used in a previous study.^11^

## Results

### Screening results

After screening the titles and abstracts, 441 of 482 articles were excluded. After full-text screening, we excluded articles in which an SNS was not used (*n* = 8), which focused on the development of system (*n* = 7), which described the actual states of SNSs (*n* = 2), and in which the study subjects were not mothers (*n* = 1). Finally, 19 articles were included in this study (Fig. 1).^25–43^

### Characteristics of the selected studies

The characteristics of the included studies are shown in Table 1. The country with the highest number of reported cases was the United States (*n* = 6). The common attribute of mothers was pregnancy (*n* = 5). The SNS most frequently used for intervention was Facebook (*n* = 13). The most common support target was breastfeeding (*n* = 6). Most study designs were based on intervention (*n* = 15). Most studies indicated the positive effect of SNS use on the support target (*n* = 15).

**Table 1.** Characteristics of the Included Studies in This Review

Authors	Year	Country	Attributes of mothers	Number	SNS type	Support target	Effect	Study design
Wallis et al.^25^	2021	AUS	None in particular	120	Facebook	Body image	E	I
Morse and Brown^26^	2021	UK	Local group	2228	Facebook	Breastfeeding	E	CS
Hui et al.^27^	2021	CAN	Indigenous women	125	Facebook	Prenatal education	E	I
Liu et al.^28^	2021	CHN	None in particular	125	WeChat	Mental health	E	I
Chatwin et a.^29^	2021	UK	Pregnant women	156	Facebook	Antenatal support	E	CS
Dumas et al.^30^	2020	CAN	None in particular	84	Facebook	Feeding behavior	NE	I
Dauphin et al.^31^	2020	USA	Pregnant women	288	Facebook	Breastfeeding	E	I
McCarthy et al.^32^	2020	UK	Pregnant women	31	Facebook	Information provision	E	I
Dumas et al.^33^	2020	CAN	None in particular	84	Blog	Feeding behavior	NE	I
Dumas et al.^34^	2020	CAN	None in particular	84	Blog	Feeding behavior	NE	I
Williams et al.^35^	2019	USA	Pregnant, Hispanic women	23	Facebook	Body weight	E	I
Cavalcanti et al.^36^	2019	BRA	Women after childbirth	251	Facebook	Breastfeeding	E	I
Wright et al.^37^	2019	USA	None in particular	798	Blog	Information provision	E	I
Boyd et al.^38^	2019	USA	Depression, postpartum, ethnic minority	24	Facebook	Parenting intervention	E	I
Daley et al.^39^	2018	USA	Pregnant women	1093	Original website	Vaccine hesitancy	E	I
Bridges et al.^40^	2018	AUS	None in particular	1846	Facebook	Breastfeeding	E	CS
Niela-Vilén et al.^41^	2016	FIN	None in particular	124	Facebook	Breastfeeding	NE	I
Bahkali et al.^42^	2015	SAU	None in particular	484	Twitter	Breastfeeding	E	CS
Herring et al.^43^	2014	USA	Urban, low-income	18	Facebook	Body weight	E	I

CS, cross-sectional study; E, effective; I, intervention study; NE, not effective; SNS, social networking service.

After extracting issues associated with using SNSs to support mothers from the 19 articles, three major issues were detected (Table 2): (1) issues on the management side, (2) issues on the user side, and (3) social and environmental issues. As listed in Table 2, first, the management side issues included the following nodes: information security (*n* = 10), securing and training human resources (*n* = 9), validity of the system (*n* = 5), provision of authoritative information (*n* = 3), quick response (*n* = 2), and scope of the control of the system (*n* = 1). These issues were largely related to the safe use of SNSs and the need for professional teams to support mothers.

**Table 2.** Details of Issues

Issues	Topics described in the articles	Total number of articles
Management side	Information security^25,27,29,32,35,36,38,39,40,41^ Securing and training human resources^26,29,31,34,36,40–43^ Validity of the system^25,27,29,32,35^ Provision of authoritative information^29,32,37^ Quick response^27,32^ Scope of control of system^25^	15
User side	Busyness of mothers^25,30,33,34,41^ The existence of other support routes^29,33,34^ The psychological factors of mothers^25,27,28,41^	7
Social and environment	Social and cultural limitations^30,36^ Communication environment-related limitations^25^	3

Reference numbers are shown on the top right for each topic.

Second, the user side issues included the following nodes: busyness of mothers (*n* = 5), existence of other support routes (*n* = 3), and psychological factors of mothers (*n* = 3). These issues were related to the situation of mothers with pregnancy, childbirth, and childcare. Third, the social and environmental issues included the following nodes: social and cultural limitations (*n* = 2) and communication environment-related limitations (*n* = 1). The issues concretely expressed that geographically, there are areas with no or slow Wi-Fi service^25^ and that families in low-income countries encounter some problems in joining SNSs or obtaining communication devices.^36^

## Discussion

This study demonstrated three major issues associated with using SNSs as a tool to support mothers during a childbirth and childcare period; that is, the management side issues, user side issues, as well as social and environmental issues. This is the first review to summarize the issues associated with using SNSs for the support of mothers in the medical field. These issues can help SNSs become a more powerful tool for supporting mothers.

In this review, information security and/or securing and training human resources were the most frequently described topics. Information about pregnancy, childbirth, and childcare is extremely personal and private. The information requires sensitive treatment. When such mothers use SNSs, it would be crucial to protect their information. This seems to partly explain the fact that the information security was one of the most frequently described topics. We observed that Facebook was the most frequently used SNS in this review; even though Facebook can be used to create a closed group, information security remains the greatest concern. The information literacy of mothers who join and/or access SNSs is also debatable, although no articles about information literacy were found in this review.

In relation to such literacy, as another topic that was frequently described, securing and training human resources are mandatory when mothers use SNSs. Although mothers require correct medical information on pregnancy, childbirth, and childcare,^26,29,31,34,36,40–43^ incorrect information can be spread; thus, the involvement of human resources with professional knowledge in social networking sites that provide support for mothers is expected.^32^ In fact, we could observe that the individuals providing support included licensed midwives,^29^ trained peer breast-training counselors,^40^ and female obstetricians/gynecologists with more than 25 years of practice.^42^ However, securing and training such professional human resources are not commonly present worldwide.

Among the user side issues, busyness of mothers was the most frequently described topic.^25,30,33,34,41^ It is thought to be reasonable because of the consensus that mothers are busy from the time of pregnancy to the childbirth and childcare periods. The noteworthy point is that mothers stopped or declined to participate in SNSs because of their busy schedules.^30,33,34^ In this review, most studies found positive effects of SNS use on mothers' health conditions (Table 1); thus, we think that mothers stopping the use of SNSs or declining to use SNSs represent a serious problem. To address this, it is of the utmost importance to design SNSs in an easy way to suit the mothers' schedules.^25^ In addition, support is required to make the use of SNSs convenient for mothers.

Social and environmental issues included economic difficulties in obtaining access to SNSs and vulnerable communication environments (*e.g.,* Wi-Fi service).^25,30,36,44^ These social and environmental issues are faced by many mothers all over the world. To overcome this, social support is needed to bridge the digital divide.^45^ This cannot be realized by efforts in the medical field alone; thus, collaboration with the government is necessary.

The present study was associated with some limitations. First, the areas where SNSs support has been used or is being considered in the articles are mostly developed countries (mainly in Europe and the United States) and such use has not been considered in Asia or developing countries. Therefore, research targeting Asia and developing countries is needed. Second, most of the research subjects were pregnant and breastfeeding women, with few studies on mothers with specific diseases or chronic illnesses. If a mother's problems are disease-specific, her SNS use could be different from that of non-diseased subjects. Third, there were no specific studies to compare the various methods of SNS use that provide effective support for mothers. This will be future work.

## Conclusions

This study revealed three major issues with several topics associated with using SNSs as a tool to support mothers. These issues can provide focused hints for the future use of SNSs to support mothers, even while SNSs are simply adopted to supplement various support methods. Further research on this topic is needed.

## Abbreviations Used

CS: cross-sectional study
E: effective
I: intervention study
NE: not effective
SNS: social networking service
